# Comprehensive understanding of B7 family in gastric cancer: expression profile, association with clinicopathological parameters and downstream targets

**DOI:** 10.7150/ijbs.39769

**Published:** 2020-01-01

**Authors:** Dan Li, Shixin Xiang, Jing Shen, Mingtao Xiao, Yueshui Zhao, Xu Wu, Fukuan Du, Huijiao Ji, Mingxing Li, Qijie Zhao, Parham Jabbarzadeh Kaboli, Xiao Yang, Zhangang Xiao, Bo Qin, Qinglian Wen

**Affiliations:** 1 Department of Oncology, The Affiliated Hospital of Southwest Medical University, Luzhou 646000, China.; 2 Laboratory of Molecular Pharmacology, Department of Pharmacology, School of Pharmacy, Southwest Medical University, Luzhou, 646000, Sichuan, PR China.; 3 South Sichuan Institute of Translational Medicine, Luzhou, 646000, Sichuan, PR China.; 4 Shenzhen Aier Aye Hospital, Shenzhen, 518032, Guangdong, PR China

**Keywords:** B7 family members, gastric cancer, bioinformatics, PI3K-AKT signaling pathway

## Abstract

**Objectives:** B7 family members were identified as co-stimulators or co-inhibitors of the immune response and played important roles in cancer immunotherapy; however, their dysregulation in gastric cancer is still unclear.

**Methods:** Data were obtained from TCGA and GTEX database. B7 mutations, association with DNA methylation and affected proteins were analyzed in cBioportal. Kyoto Encyclopedia of Genes and Genomes (KEGG) enrichment analysis and Gene Ontology (GO) project was studied by DAVID to find the downstream signaling pathway and important metabolic process, respectively. Protein-protein interaction network was analyzed in STRING and Cytoscape. A total of 160 paired specimens in tissue microarray from patients with gastric cancer were used to detect the expression levels of seven B7 family members via immunohistochemical analysis.

**Results:** Bioinformatics studies revealed dysregulation of B7 members in gastric cancer. Gene and protein alteration were found in B7 family members. Furthermore, DNA methylation and gene alteration may be both involved in B7 member dysregulation in gastric cancer. Importantly, the high expression of B7-H6 is associated with good overall patient survival. B7 family members primarily affect the EGFR tyrosine kinase inhibitor resistance signaling pathway in gastric cancer and TP53 may be an important target of the family. The low expression of B7-1 and high expression of B7-H3 and B7-H7 were validated by IHC staining.

**Conclusions:** Our results provide insight into B7 family member expression in gastric cancer and stress their importance in stomach tumorigenesis, which may be beneficial for designing future cancer treatments.

## Introduction

Gastrointestinal (GI) cancer is one of the most well-known cancers due to its high lethality and poor prognosis, which are affected by environment, dietary habits, and gender 1. The complex carcinogenic mechanisms of GI cancer remain unclear; however, variables, such as genetic background, smoking, dietary habits, and geographical environment, are important factors in the progression of GI cancer 2. Gastric cancer, one type of GI cancer, accounts for approximately 8.6% of newly diagnosed cancer cases worldwide, making it the second highest cause of cancer deaths 3. Since gastric cancer is generally diagnosed in the advanced stages, treatments are often ineffective 4. Common treatments include surgery, chemotherapy, and radiotherapy; however, these methods can cause significant physiological distress, economic burden, and poor quality of life 5. Recently, immunotherapy has been used for cancer treatment, and has potential as a successful tumor therapy and for the prevention of tumor recurrence 6.

B7 family is an important family of costimulatory molecules that promotes or inhibits T cell proliferation and cytokine production, and also plays an important regulatory role in B cell activation and antibody production. Over the past decade, ten members of the B7 gene family have been identified, including B7-1, B7-2, B7-DC, B7-H1, B7-H2, B7-H3, B7-H4, B7-H5, B7-H6 and B7-H7. Each member contains at least 15% conjunct amino acid sequences, which are expressed by antigen-presenting cells (APCs) or tumor cells 7. B7 family members can be divided into three groups: group I, which includes B7-1/B7-2/CD28/CTLA4 and B7-H2; group II, which contains PD-L1/PD-L2/PD-1, and group III, which consists of B7-H3, B7-H4, and B7-H5. B7 family ligands and receptors play important roles in T-cell regulation of the immune system 8. Several studies have indicated that B7 family members are overexpressed in tumors and are associated with pathogenesis and malignancy due to their immunological functions in tumor-bearing hosts, as in lung and hematologic cancers 9-11. Moreover, soluble B7 family ligands have also been detected in the sera of tumor patients; soluble B7-H3 and soluble B7-H4 have proven to be prognostic biomarkers in ovarian and renal cancer 12. In addition, B7-H1 and B7-H4 were shown to be positively correlated with the depth of tumor invasion, lymph node metastasis, and tumor stage, and are considered to be negative prognostic factors in gastric cancer 13. B7-H4 is also positively associated with poor prognosis in gastric cancer 14. B7-H2 was shown to be significantly related to the occurrence of gastric cancer through the SNP rs4819388, which disrupts the regulatory role of miR-24 on B7-H2 expression and promotes the occurrence of gastric cancer 15. Another study demonstrated that the aberrant expression of B7-H3 promoted cell migration and invasion in gastric cancer patients compared to the controls 16. Although B7 family members were found to be related to the progression of GI cancer, the expression patterns of each member in GI tumors, especially in gastric cancer, are still not clear. Meanwhile, there is little research progress in the biological function and mechanism of B7 family members in gastric cancer. In this study, we explored the relationship between the expression levels of B7 family members in gastric cancer and show mutations and survival analysis of B7 family members, and association of B7 family with clinicopathological parameters in gastric cancer. Finally, we conducted Kyoto Encyclopedia of Genes and Genomes (KEGG) enrichment analysis and Gene ontology (GO) analysis of B7 family, and finally we obtained one key protein for further analysis. We hope that these studies will help early detection and treatment strategies for patients with gastric cancer.

## Results

### The dysregulation of B7 family members in gastric cancer

**Figure 1:**
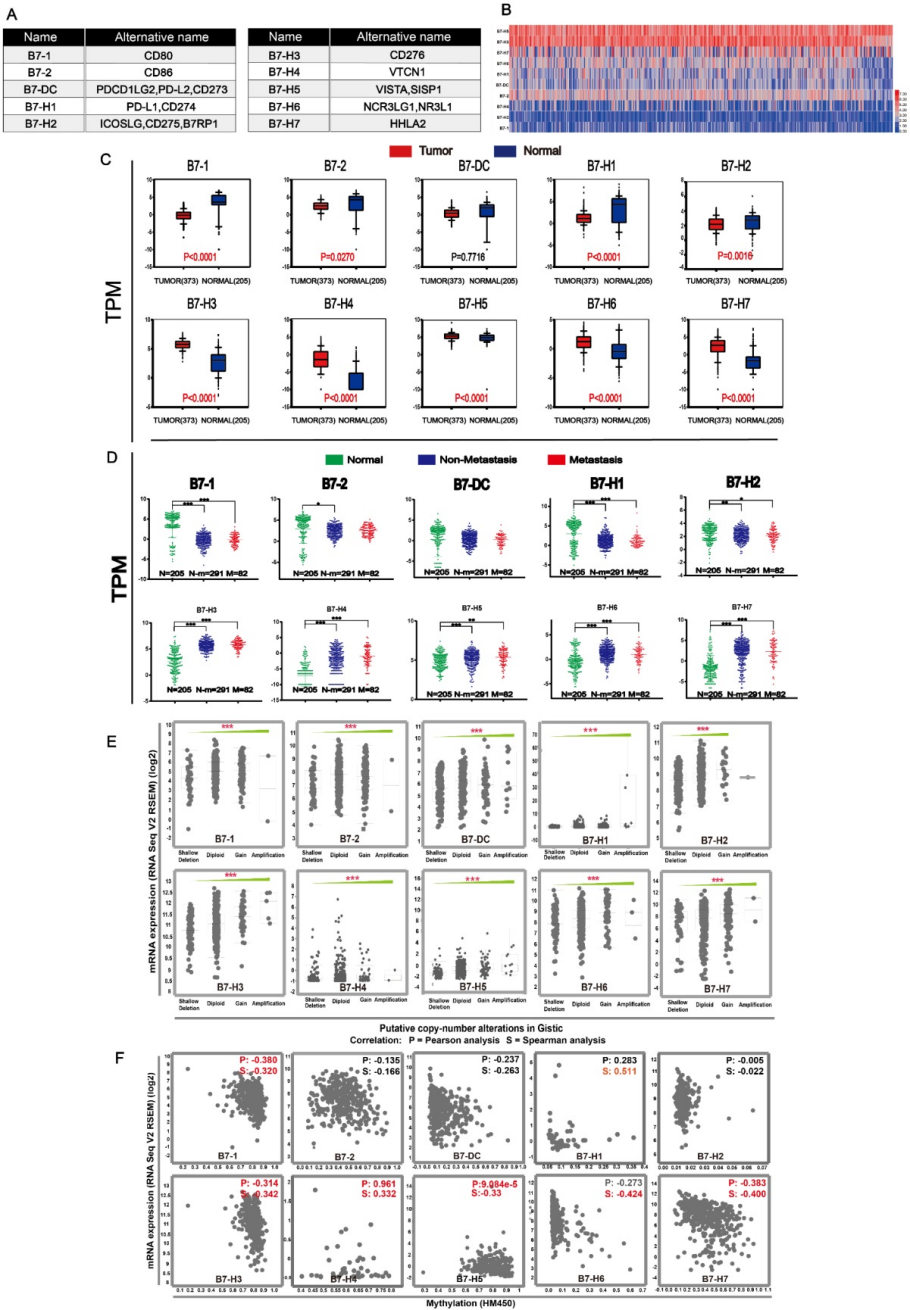
** Expression level of B7 family members in gastric cancer.** A. Nomenclature of each B7 family member. B. Heat map showing expression levels of B7 family genes between cancer and normal samples based on RNA sequencing data from TCGA database. Data were collected from tumor (n = 373) and normal tissues (n = 32). C. RNA sequencing data from TCGA and GTEX database of 373 cancer patients and 203 healthy people were used to analyze the expression levels of B7 family genes in gastric cancer. D. RNA sequencing data from TCGA and GTEX database were used to evaluate the expression levels of B7 family members in 203 normal people, 291 non-metastasis patients and 82 metastasis patients. E. Association of B7 family member mRNA expression with gene alteration. F. Association of B7 family member mRNA expression with promotor methylation. (* P < 0.05,** P < 0.01, and *** P < 0.001).

The ten members of the B7 family were shown in **Fig. 1A**. We compared the expression levels of B7 members in normal and tumor samples (**Fig. 1B-C**). Results showed that B7-H3, B7-H4, B7-H5, B7-H6 and B7-H7 were significantly upregulated, while B7-1, B7-2, B7-H1 and B7-H2 were significantly downregulated in gastric cancer patients. (**Fig. 1C**). At the same time, we also compared the expression levels between normal, non-metastatic and metastatic gastric cancer (**Fig. 1D**). Although there was significant difference between normal and cancer samples, there is no significance between non-metastatic cancer and metastatic gastric cancer. Then, we examined the mRNA expression levels of B7 family members and their relationship to DNA methylation, copy number alterations to obtain an overview of the dysregulation mechanism of B7 family members in gastric cancer (**Fig. 1E-F**). Gene alterations, including diploid, copy number gain, and gene amplification, were associated with the increased expression of B7 members (**Fig. 1E**). Moreover, there was a negative correlation between mRNA expression and promotor methylation for B7-1, B7-H3, B7-H4, B7-H5, B7-H6, and B7-H7 (**Fig. 1F**), suggesting promotor methylation might also be involved in B7 members' dysregulation.

### Gene and protein alteration of the B7 family members

**Figure 2:**
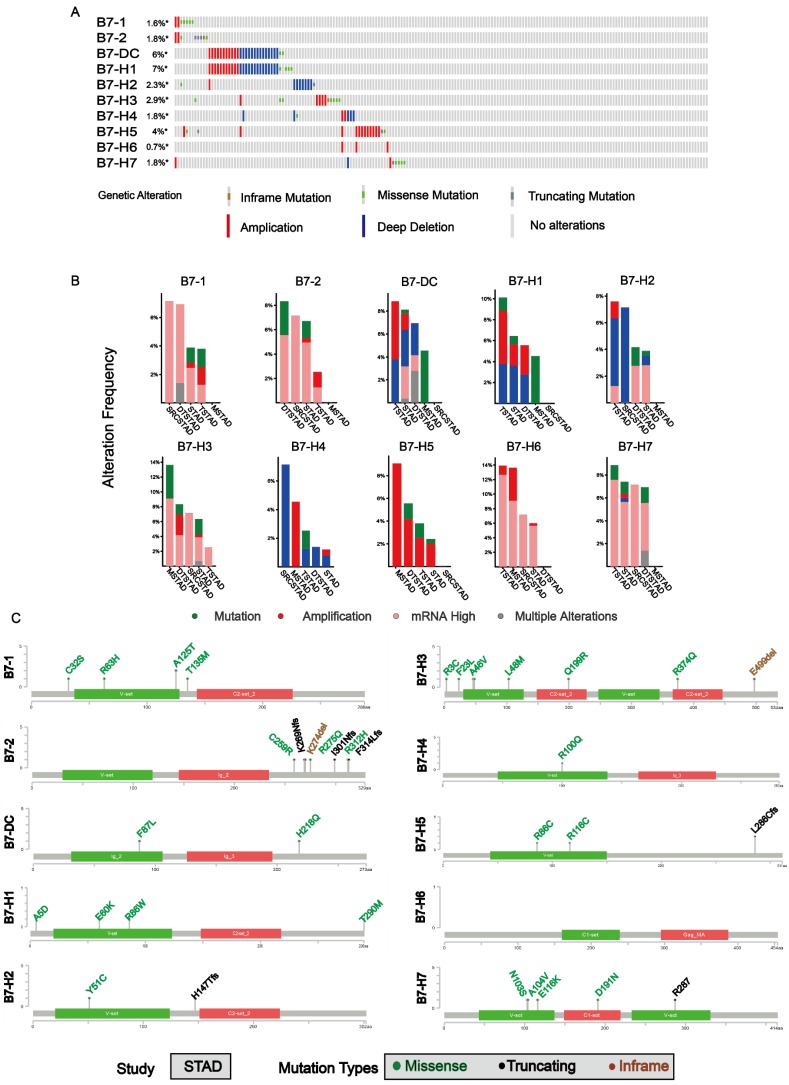
** Gene and protein alteration of B7 family members in gastric cancer.** A. The mutation rate of B7 family members in gastric cancer. B. Alteration frequency of B7 family genes in gastric cancer, including mutations, amplification, mRNA overexpression and multiple changes were analyzed from the TCGA database and studied in cBioPortal. 478 gastric cancer samples were used for this analysis, and about 14% samples (66/478) showed B7 family alteration. C. Changes in protein structure of B7 family members. SRCSTAD: Signet ring cell carcinoma of the stomach; Tubular stomach adenocarcinoma: TSTAD; Stomach adenocarcinoma: STAD; Diffuse type stomach adenocarcinoma: DTSTAD; Mucinous stomach adenocarcinoma: MSTAD

In order to analyze mutations of B7 family member in gastric cancer, cBioPortal for Cancer Genomic was used. Overall, about 16% samples (63/393) had genetic changes is analyzed in gastric cancer, as shown in **Fig. 2A**. The mutation ratio of B7-DC and B7-H1 were relatively higher, 6% and 7% respectively. Meanwhile, the mutation frequency of B7 family member in different types of gastric cancer was shown (**Fig. 2B**). mRNA overexpression was frequently seen in B7 family members. The frequency of mRNA overexpression was high in B7-1, B7-2, B7-H3, B7-H6 and B7-H7 and B7-H5 showed relatively higher copy number amplification. Changes in protein structure of B7 family member were shown in **Fig. 2C**. There were more mutation sites in B7-2 and B7-H3 than in other members. The protein domains of B7 family members were shown in **Supplementary Fig. 1**.

### The association of B7 family with clinicopathological parameters and patient survival in gastric cancer

**Figure 3:**
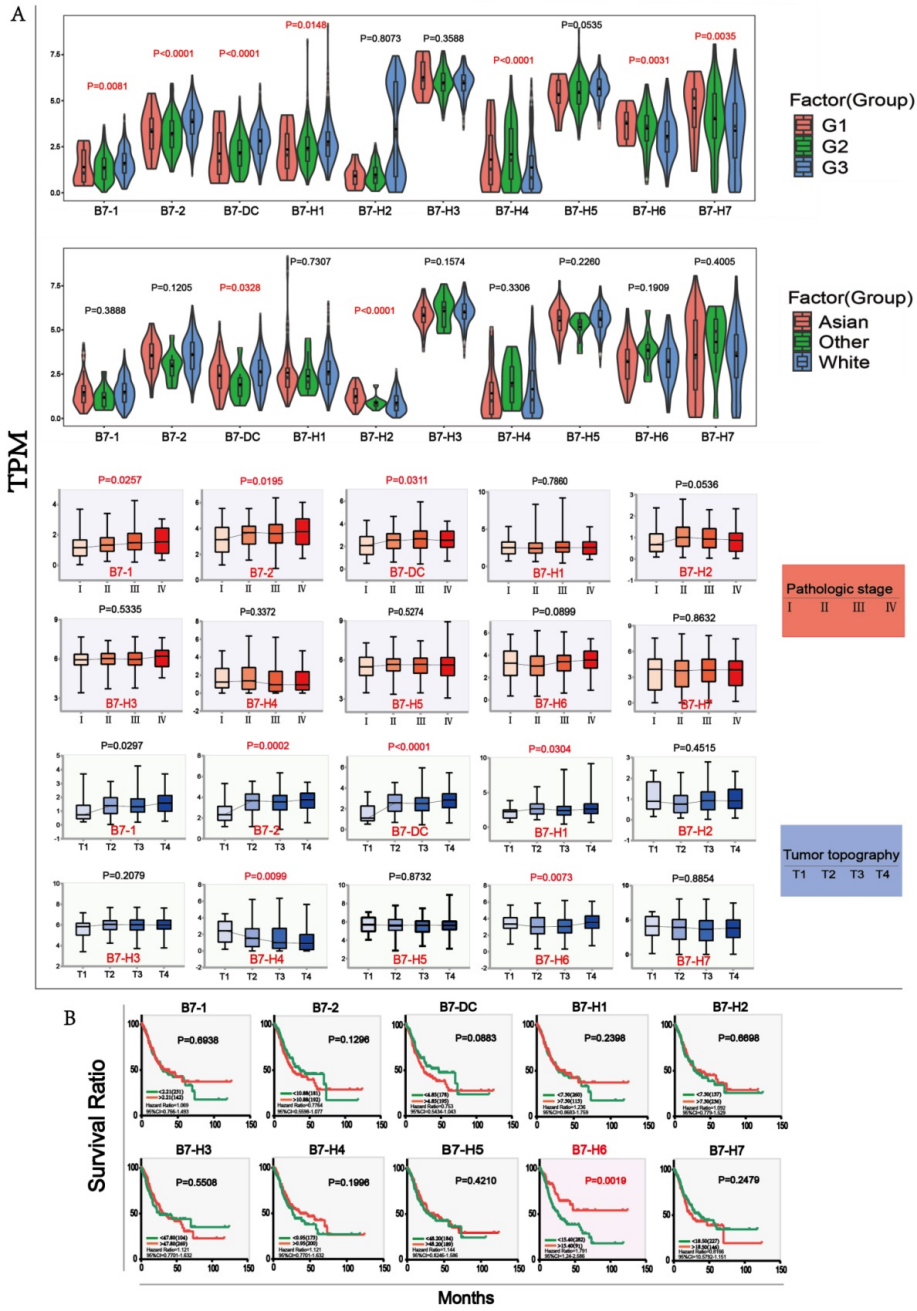
** The association analysis of B7 family with clinical parameters and Kaplan-Meier survival analysis in gastric cancer.** A. Clinical parameter analysis includes race (Asian, White, Others), stage of tumor differentiation (G1, G2 and G3), pathologic stage (Stage I, Stage II, Stage III, Stage IV) and tumor topography (T1, T2, T3, T4) by one-way ANOVA. B. Kaplan-Meier survival curves of B7 family genes in gastric cancer based on the expression level. (* P < 0.05**, P < 0.01, and *** P < 0.001).

We analyzed the association of B7 family expression with overall survival status (Dead; Alive), gender (Male; Female), cancer status (Tumor; Normal), race (Asian; White; Others), tumor differentiation grade (G1; G2; G3), pathological stage (Stage I; Stage II; Stage III; Stage IV), tumor topography (T1; T2; T3; T4) and lymph node(N1; N2; N3; N4) through chi-square test as shown in **Table 1**. The results showed that the expression of some B7 family member was significantly associated with tumor differentiation grade, pathological stage, race and tumor topography. The relationship between the four clinical parameters and expression levels of B7 family members was shown in **Fig. 3A**. For tumor differentiation grade, B7-1, B7-DC and B7-H1 gradually increased from G1 to G3. Conversely, B7-H6 and B7-H7 gradually decreased. For pathological stage, the expression of B7-1, B7-2 and B7-DC were elevated from pathological stage I to IV. Moreover, there are significant differences between B7-DC and B7-H2 in race. For tumor topography, the relationship between the expression of B7-2, B7-DC, B7-H1, B7-H4, B7-H6 and the extent of the primary tumor had significant differences. Next, we combined the data of gene expression and clinical information in the TCGA database to evaluate the influence of B7 family member expression on survival rate in patients with gastric cancer. Kaplan-Meier analysis showed high expression of B7-H6 was significantly associated with longer OS in gastric patients (**Fig. 3B**).

### Pathway and GO analysis of B7 family members

**Figure 4:**
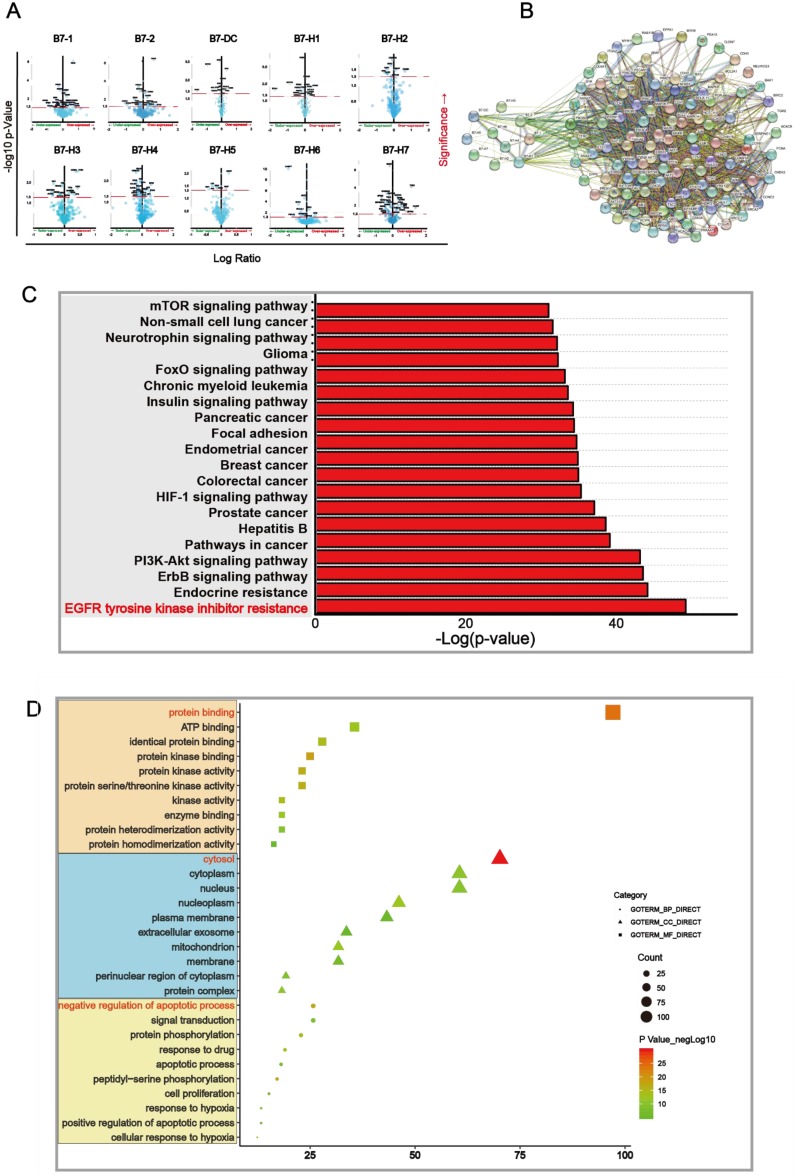
** Main signaling pathway affected by B7 family members.** A. Volcano plot was drawn to identify differential proteins affected by B7 members analyzed using Reverse Phase Protein Arrays (RPPA) in cBioPortal. The Y axis is the value of fold change of expression level is based on logarithmic ratio (mean of changed expression/mean of unchanged expression). -log10 (p-value)>1.30 is considered to be a significant difference. B. Multicenter protein-protein interaction (PPI) network analysis between B7 family member and differential proteins in STRING database. The stronger the interaction between the two proteins, the thicker the lines. C. Prediction of downstream pathways related to B7 family genes alterations was analyzed by KEGG pathway analysis via DAVID. D. Bubble chart of the GO (Gene Ontology) enrichment analysis of the B7 family was analyzed via DAVID. Gene count and P values were considered to obtain important metabolic process.

We further found out differential proteins that were altered upon B7 family member mutation. Proteins which have significant changes (p < 0.05) were shown in the volcano map (**Fig. 4A**) and were used for further analysis. We mapped Protein-Protein Interaction Networks for further understanding the interaction between these differential proteins and B7 family member. (**Fig. 4B**). In order to identify which pathway the differential protein is mainly enriched in, we analyzed KEGG proteins enrichment by DAVID. According to the p value, EGFR tyrosine kinase inhibitor resistance is considered to be the most important downstream signaling pathway for members of the B7 family in gastric cancer (**Fig. 4C**). Finally, we analyzed the 105 differential proteins by GO terms biological process enriched via DAVID. The result showed that these differential proteins mainly play an important role in the regulation of protein binding, cytosol, negative regulation of apoptotic process in biological process, cellular component and molecular function respectively (**Fig. 4D**).

### TP53 is closely related to B7 family members in gastric cancer

**Figure 5:**
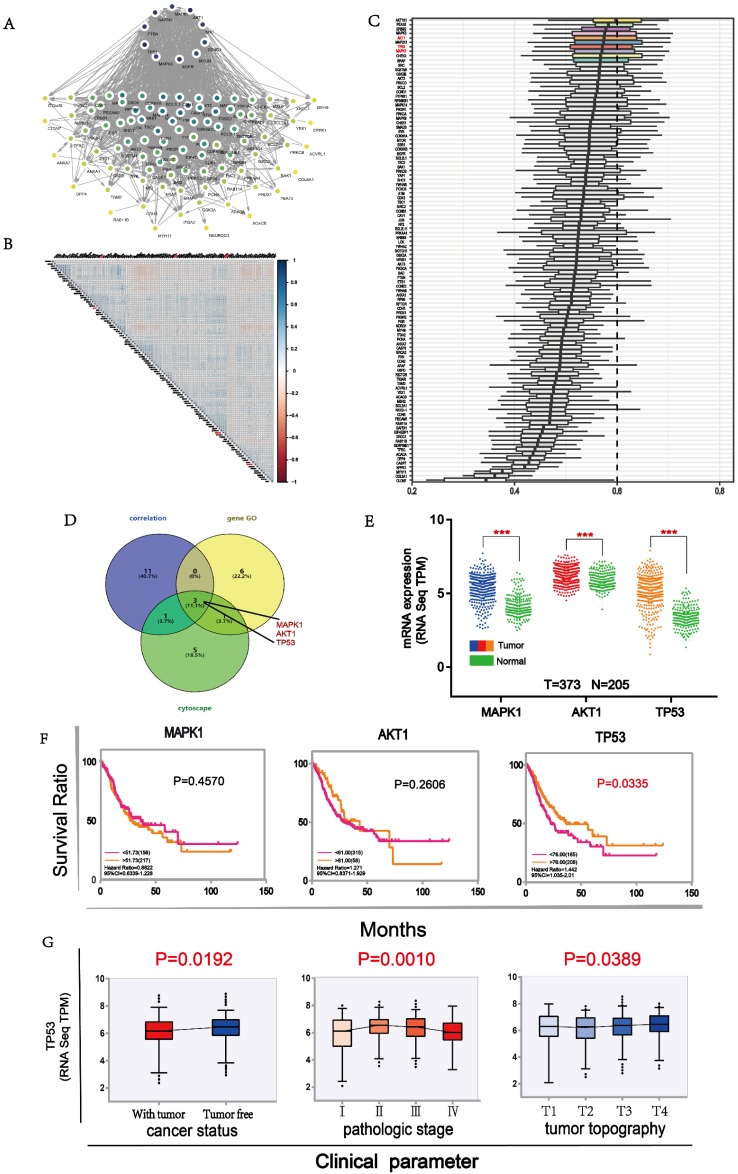
** Main targets of B7 members.** A. The protein interaction network diagram of differential proteins by cytoscape. Ten proteins in black color were strongly connected to other proteins. B. Co-expression analysis of B7 differential proteins. The color intensity reflects the reliability of co-expression. C. GO analysis of differential proteins. Ten proteins were shown to be important in biological processes, cell components, and molecular functions. D. Venn diagram was used to screen important downstream proteins by 3 methods. MAPK1, AKT1, TP53 were predicted to be major downstream targets. E. The comparison of expression level of MAPK1, AKT1 and TP53 between tumor and normal samples. F. Survival analysis of MAPK1, AKT1 and TP53 in gastric cancer. G. Clinical parameters analysis of TP53 in gastric cancer.

To identify the most important proteins of the 105 differential proteins, we conducted further analysis. First, we mapped Protein-Protein Interaction Networks through cytoscape for understanding the interaction between these differential proteins. Ten proteins demonstrated stronger interaction among all proteins (**Fig. 5A**). Then, we used the expression level of all differential proteins to conduct a correlation analysis and selected 15 proteins with good correlation with other proteins (**Fig. 5B**). Next, we compared the importance of functional annotations of different proteins. The results showed that the first ten proteins were strongly involved in the three major categories of GO (Gene Ontology), including biological processes, cell components, and molecular functions. (**Fig. 5C**). Finally, three proteins, namely MAPK1, AKT1 and TP53, were predicted to be the most important downstream targets by the above 3 approaches (**Fig. 5D**). Their expressions were significantly correlated with some B7 members (**Supplementary Fig. 2**). In order to understand the expression of these proteins in gastric cancer, we compared the expression levels of them between tumor and normal samples. The results showed that the expression levels of these proteins in cancer patients were significantly higher than in normal patients (**Fig. 5E**). Moreover, the high expression of TP53 was significantly associated with longer OS in gastric patients (**Fig. 5F**). Finally, we conducted clinical parameters analysis of TP53 in gastric cancer. Results showed that the expression level of TP53 was significantly different in cancer status, pathologic stage and tumor topography (**Fig. 5G**), with its expression lower in patients with tumor than tumor free. In conclusion, we believe that TP53 may be an important downstream target of B7 family members in gastric cancer.

### Verification of B7 family expression level in patient samples

**Figure 6:**
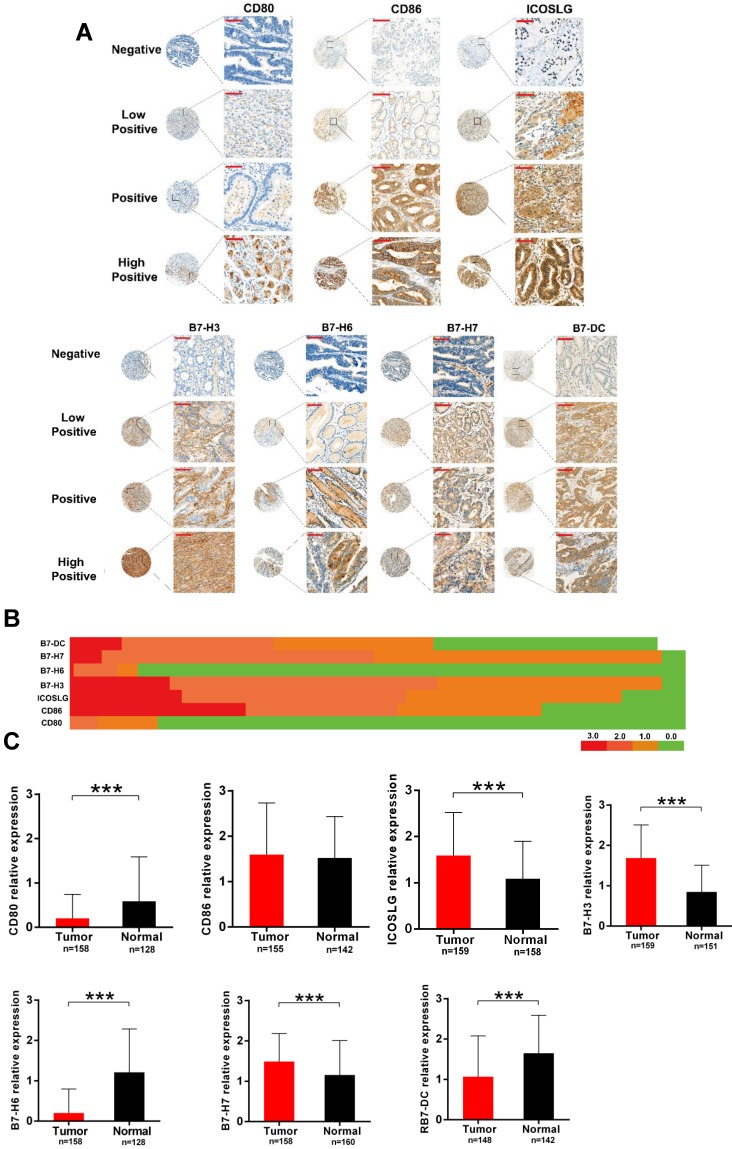
** Verification of B7 family expression in gastric cancer samples.** A. Representative immunohistochemical (IHC) staining showing B7 family expression in tumor tissues. Negative (blue, Score = 0; cases: CD80 135/158, CD86 36/155, ICOSLG 20/159, B7-H3 10/159, B7-H6 140/158, B7-H7 9/158, B7-DC 56/148), Weak (buff, Score = 1; cases: CD80 15/158, CD86 36/155, ICOSLG 54/159, B7-H3 56/159, B7-H6 5/158, B7-H7 72/158, B7-DC 40/148), Moderate (yellow-brown, Score = 2; cases: CD80 7/158, CD86 38/155, ICOSLG 56/159, B7-H3 67/159, B7-H6 12/158, B7-H7 68/158, B7-DC 38/148), and Strong (brown, Score = 3; cases: CD80 1/158, CD86 45/155, ICOSLG 29/159, B7-H3 26/159, B7-H6 1/158, B7-H7 9/158, B7-DC 14/148); (magnification: x400, Scar bar = 50 μm). B. Heatmap clustering showing the expression level of B7 family molecules in gastric cancer samples. C. Quantification of B7 family member levels in paired gastric cancer and adjacent normal tissues by IHC staining. (***p < 0.001).

To verify the results of bioinformatics analysis, we performed immunohistochemical (IHC) staining on tissue microarray slides containing 160 gastric cancer tissues. **Fig. 6A** represents the staining patterns of B7 family members in gastric cancers. In all of the tissues, the staining of B7 family members was observed in the cytoplasm or cell membrane but not the nucleus. Brown, yellow-brown, or buff presented positive staining cells as strong, moderate, and weak, respectively. We then performed heatmap clustering analysis for seven of the B7 family members in gastric cancer samples (**Fig. 6B**). Results showed higher staining of CD86 (118/157, 75%), ICOSLG (122/157, 77%), B7-H3 (138/157, 88%), B7-H7 (138/157, 88%), and B7-DC (104/157, 66%) and overall lower staining of CD80 and B7-H6 in gastric cancer tissues. The expression levels of B7 family members between tumor and normal tissues were shown in **Fig. 6C**. Seven B7 family molecules were unequivocally expressed in all stomach tumor samples and paired non-tumor tissues from the same patient. Moreover, ICOSLG (89.6%, 138/154; *P* < 0.001), B7-H3 (96.1%, 148/154; *P* < 0.001) and B7-H7 (96.1%, 148/154; *P* < 0.001) were significantly upregulated in tumors. In contrast, CD80 (14.2%, 22/154; *P* < 0.001), B7-H6 (11%, 17/154; *P* <0.001) and B7-DC (59%, 91/154; *P* < 0.001) were significantly downregulated in tumors, while CD86 (76.6%, 118/154; *P* = 0.54) was upregulated but not significantly.

## Discussion

The functions of individual immune system components in different physiological and pathological states are regulated by the functions of opposing factors. The dysregulation of the immune system influences tumor T cell immune activity in the tumor microenvironment, and may accelerate tumor progression, metastasis, and malignancy 17. The innate and adaptive immune systems play important roles in inhibiting tumor progression through T cell-mediated anti-tumor immune responses 18. It is well known that B7 family members are involved in immune checkpoints and tumor angiogenesis 19. The suppression of anti-tumor immune responses is a distinguishing feature of tumorigenesis. B7 co-stimulatory and co-inhibitory family members are involved in this process and have crucial functions in the progress of malignancies, thus they are studied as potential targets of immunotherapeutic strategies for human cancer treatment 9. B7 family members and their receptors, CD28 family members, play key roles in the regulation of the T cell response 20 and are mainly regarded as secondary signals, in cooperation with the first signals in modulating T cell response. Several B7-CD28 family members have been proven to participate in T cell activation and tolerance in peripheral tissues, including the inhibition of the immune response through the suppression of T cell functions, the regulation of cytokine production, and the stimulation of CD4+ T cell proliferation in synergy with other proteins 21. To date, only a few studies have mentioned the roles of B7 family members, such as B7-1, B7-2, B7-H3, B7-H4, and B7-H6, in gastric cancer 13,22-24. The expression patterns and downstream signaling pathway of B7 family members in gastric cancer are not well illustrated. Thus, we performed bioinformatics analysis to determine the regulation and expression patterns of B7 family members in gastric cancer, and verified the results by experiments.

In this study, first, we obtained data from the TCGA database to compare the expression levels of B7 family members with heatmap (**Fig. 1B**). At the same time, we compare the expression levels in normal and tumor samples using TCGA and GTEX database in box plot. Results demonstrated that B7-H3, B7-H4, B7-H5, B7-H6 and B7-H7 were significantly upregulated in gastric cancer, on the contrary, B7-1, B7-2, B7-H1 and B7-H2 was significantly downregulated (**Fig. 1C**). Moreover, we performed IHC staining on tissue microarray slides containing 160 gastric cancer tissues. Results showed higher staining of B7-H2, B7-H3, B7-H7 and lower staining of B7-1, B7-H6 and B7-DC in gastric cancer tissues, which is partly consistent with the bioinformatics result. However, the result for the B7-1 and B7-H6 is the opposite (**Fig. 6B**). Overexpression of B7-H3 in gastric cancer has been reported in different studies and predicts poor patient survival 16,25. Previous reports suggest that CD80 exhibits anti-tumor functions and is a co-stimulatory factor in the induction of cytotoxic T lymphocytes (CTL) in the suppression of stomach tumor metastasis 26-28. Decreased expression of CD80 was found in gastric cancer, which is consistent with our experimental results, and gene therapy with CD80 inhibits metastasis 24,27,29. B7-H6 has been demonstrated to be overexpressed in gastric cancer 30. And B7-DC was reported to be highly expressed in EBV-associated gastric cancer 31. Moreover, previous studies revealed that CD86 is highly expressed in metastatic gastric carcinoma 24 and the high expression of B7-H3 is commonly found in different cancers and predicts poor patient survival 32-34. Therefore, enlargement of the patient sample size and comprehensive study are required to further elucidate the expression and functions of B7 family members in gastric cancer. Next, we analyzed the difference in the expression levels of normal, non-metastatic and metastatic gastric cancers (**Fig. 1D**). The results showed that there is no significant difference between non-metastatic cancer and metastatic gastric cancer, suggesting B7 family was not strongly involved in gastric tumor metastasis. Further analysis revealed that DNA methylation and gene alteration may both participated in the dysregulation of B7 members (**Fig. 1E-F**).

Then, we analyzed the mutation ratio (**Fig. 2A**), mutation frequency (**Fig. 2B**) and changes in protein structure (**Fig. 2C**) of B7 family members in gastric cancer, and found that some genes showed higher mutations, such as B7-DC and B7-H1. Meanwhile, The relationship between B7 family member expression and patient clinicopathological features has been investigated in HCC, in which B7-H1/H4 were associated with serosa invasion, lymph node metastasis, and tumor stage, while B7-H3 was associated with clinical stage and distant metastasis 13,35, and B7-H6 was significantly associated with a higher differentiation 22. However, the relationship between B7 family member expression levels and the gastric cancer clinicopathological parameters has rarely been studied. Thus, we also examined the expression levels of these B7 members with clinicopathological parameters (**Fig. 3A**). Results showed that except for B7-H2, B7-H3, B7-H5, other genes were significantly associated with tumor differentiation grades, while the expression levels of B7-1, B7-2, and B7-DC were significantly different for the pathological stage. Besides, there are significant differences between B7-DC and B7-H2 for race, and tumor topography was also significantly different for B7-2, B7-DC, B7-H1, B7-H4, and B7-H6. These results indicate that members of the B7 family have important regulatory roles in gastric cancer.

Furthermore, to explore whether the expression of B7 family members is an independent prognostic factor for gastric cancer, Kaplan-Meier analysis was performed and result showed that only B7-H6 was significant related to overall survival (OS). Of note, the higher expression of B7-H6 predicted good patient survival (**Fig. 3B**), which is on the contrary to the report for other cancers. Previously there was a study indicating that B7-H6 was not predictive of patient survival in gastric cancer 22. Thus, the importance of B7-H6 as prognosis indicator in gastric cancer awaits further investigation. Several studies have reported that B7 family members were associated with poor survival rates in other cancers, such as B7-H3 in esophageal cancer, B7-H6 in lung cancer, and B7-H1 and B7-H4 in ovarian cancer 11,36,37.

To identify major modulators of B7 family function, we determined proteins affected by B7 expression in gastric cancer (**Fig. 4A**). Based on those proteins that are significantly different, we conducted a Kyoto Encyclopedia of Genes and Genomes (KEGG) enrichment analysis of the pathways in B7 family via DAVID. EGFR tyrosine kinase inhibitor resistance signaling pathway is a key downstream signaling pathways of B7 family in gastric cancer (**Fig. 4C**). Meanwhile, we also performed gene ontology (GO) and Protein-Protein interaction network analysis (**Fig. 4B**) on the differential proteins of B7 family members, and found that those proteins are mainly involved in the three regulation of protein binding, cytosol, negative regulation of apoptotic process. (**Fig. 4D**). Finally, in order to screen the most important proteins downstream of B7 family, we used 3 different approaches (**Fig. 5A-C**). Then, 3 common proteins from the 3 methods were selected for further analysis, namely MAPK1, ATK1 and TP53 (**Fig. 5D**). Next, we compared the expression levels and survival analysis of these three proteins respectively (**Fig. 5E**), and the results showed that high expression of TP53 had a good prognosis in gastric cancer (**Fig. 5F**). Therefore, we conducted clinical parameter analysis of TP53, further verifying the importance of TP53 in gastric cancer (**Fig. 5G**).

To our knowledge, this is the first time the expression patterns, mutations and downstream signaling pathway of B7 family members in gastric cancer have been studied. Our results confirmed the overexpression of B7-H3 and B7-H7 by both bioinformatics and experimental analysis. Collectively, our findings provide new insight into the roles of B7 family members in gastric cancer and likely have important implications for future immunotherapy in the treatment of gastric cancer.

## Materials and Methods

### Data Processing

The expression level and clinical data of B7 family members in gastric cancer were extracted from TCGA database (http://cancergenome.nih.gov) and GTEX database (https://www.gtexportal.org/home/). GISTIC 2.0 database from the cBioportal website (https://www.cbioportal.org/) was used to visualize data such as somatic mutations, copy number changes, DNA methylation and reverse phase protein arrays for further signal pathway analysis. cBioPortal data comes from multiple websites, such as the TCGA data portal (https://tcga-data.nci.nih.gov/tcga/), the ICGC data portal (http://dcc.icgc.org/), the Broad Institute's Genome Data Analysis Center (GDAC) Firehose (http://gdac.broadinstitute.org), the IGV, the University of California, Santa Cruz (UCSC) Cancer Genomics Browser and so on. The association of mRNA expression levels of B7 members with DNA methylation, copy number alterations were analyzed in cBioportal for Cancer Genomics database in gastric cancer samples (N = 408).

### Protein Structure Alteration

Lollipop of each protein structure change of gastric cancer were linked to COSMIC. The detailed mutation annotation from OncoKB, CIViC and Hotspot in different genes were displayed in different regions of the protein structure.

### R project analysis

R/Bioconductor GOsemsim and org.Hs.eg.db package was used for gene importance analysis of three types of GO, and correlation analysis of differential proteins by R package corrplot.

### Pathway Analysis

Proteomic data were collected by Reverse Phase Protein Array (RPPA) based on TCGA data from cBioportal (http://www.cbioportal.org/). For the enriched proteins, significant change in expression was determined by the standard of Log2 based ratio (μ mean altered/ μ mean unaltered) (log>0 for over-expression and log<0 for under-expression) and queried event results in p value<0.05. The -log10 p-value >1.30 proteins were selected for further downstream pathway analysis. Finally, the differential proteins were used to predict the pathway in the DAVID function annotation tool (https://david.ncifcrf.gov).

### Gene Ontology analysis

The GO (Gene Ontology) enrichment analysis of B7 family members was analyzed via DAVID function annotation tool. Gene ontology includes three parts: molecular function, biological process and cellular component. The count and P values were considered together to obtain important metabolic process.

### Protein-protein interaction network analysis

We conducted the Protein-protein interaction network analysis by using the STRING and Cytoscape software. The database of STRING is a meta resource, including both physical and functional interactions 38.STRING can be reached at http://string-db.org/. Cytoscape can be for visualizing biomedical networks consisting of proteins, genes and other types of interactions, and is one of the most popular open source software tools 39.

### Patients and tissue samples

Tumor and adjacent non-tumor tissue (160 cases) collection, informed consent and protocols are from the First Affiliated Hospital, Wannan Medicine College (Wuhu, China). Archived samples were collected from patients during 2011-2012 and the study was conducted in 2017. Each tumor sample was paired with non-tumor tissues (> 5cm from the tumor edge). The tissue samples were fixed in 10% buffered formalin and embedded in paraffin. Authors do not have access to information that could identify individual participants during or after data collection.

### Immunohistochemical staining

Pathological sections were used to mark the locations of cancer tissues and adjacent normal tissues on the corresponding wax blocks, and immune chips were constructed according to the cancer tissues and adjacent tissues. IHC was performed on the immune chip using ChemMateTM Envision/HRP technology. Briefly, after the sections were first dewaxed, endogenous peroxidase activity was blocked using H_2_O_2._ The primary antibodies for B7-1, B7-2, B7-H2, B7-H3, B7-H6, B7-H7 and B7-DC were incubated with a diluted solution, and then a secondary antibody was added and developed with diaminobenzoquinone (DAB). Finally, the slides were counterstained with hematoxylin.

B7-1, B7-2, B7-H2, B7-H3, B7-H6, B7-H7, and B7-DC positivity were assessed based on overall staining intensity and area. The staining intensity scores were as follows: Negative: 0, Weak positive: 1, Moderate positive: 2, Strong positive: 3. According to the semiquantitative counting method, antibody expression was calculated by measuring both intensity and region of staining: =0%:0; 1%< to <10%:1; 11% to <49%: 2; ≥ 50%: 3. The final score was determined by the intensity of the case, which was calculated by multiplying the area fraction. A final score of 0 was considered as a negative expression, scores of 1 to 3 were considered low to mild positive expression, and scores greater than 3 were considered to be high positive expression. All of the slides were independently evaluated by two researchers blinded to patient identities and clinical conditions.

### Statistical analysis

Statistical analysis was performed using GraphPad Prism 6 and SPSS 16.0 software. Student's t test was used to compare the difference between two groups, and one-way ANOVA was used to compare multiple groups. Overall survival was shown as a Kaplan-Meier curve, which was calculated using the log-rank test. p<0.05 was considered statistically significant.

## Supplementary Material

Supplementary figures.

## Figures and Tables

**Table 1:** B7 family's mRNA expression and clinicopathological Characteristics of gastric cancer patients in the TCGA database.

Characteristics		B7											
		High	Low	χ2	P	High	Low	χ2	P	High	Low	χ2	P
**OS status**		**B7-1**				**B7-2**				**B7-DC**			
	**Dead**	112	117	0.3565	0.5505	111	118	0.6557	0.4181	106	123	3.509	0.0610
	**Alive**	75	69			76	68			81	63		
		**B7-H1**				**B7-H2**				**B7-H3**			
	**Dead**	114	115	0.02946	0.8637	117	112	0.2176	0.6409	106	123	3.509	0.0610
	**Alive**	73	71			70	74			81	63		
		**B7-H4**				**B7-H5**				**B7-H6**			
	**Dead**	121	108	1.735	0.1877	115	114	0.001686	0.9672	117	112	0.2176	0.6409
	**Alive**	66	78			72	72			70	74		
		**B7-H7**											
	**Dead**	112	117	0.3565	0.5505								
	**Alive**	75	69										
**Gender**		**B7-1**				**B7-2**				**B7-DC**			
	**Male**	72	61	1.324	0.2499	67	66	0.0215	0.8834	70	63	0.5157	0.4727
	**Female**	115	125			119	121			117	123		
		**B7-H1**				**B7-H2**				**B7-H3**			
	**Male**	73	60	1.868	0.1717	70	63	0.5157	0.4727	65	68	0.1317	0.7167
	**Female**	114	126			117	123			122	118		
		**B7-H4**				**B7-H5**				**B7-H6**			
	**Male**	60	73	30.81	<0.0001	66	67	0.0215	0.8834	71	62	0.873	0.3501
	**Female**	127	113			121	119			116	124		
		**B7-H7**											
	**Male**	63	70	0.6324	0.4265								
	**Female**	124	116										
**Cancer status**		**B7-1**				**B7-2**				**B7-DC**			
	**Normal**	59	61	0.07221	0.7881	61	59	0.035	0.8516	63	57	0.4133	0.5203
	**Tumor**	109	106			107	108			105	110		
		**B7-H1**				**B7-H2**				**B7-H3**			
	**Normal**	57	63	0.5249	0.4687	59	61	0.07221	0.7881	65	55	1.207	0.2719
	**With Tumor**	111	104			109	106			103	112		
		**B7-H4**				**B7-H5**				**B7-H6**			
	**Normal**	63	57	0.4133	0.5203	66	54	1.76	0.1846	53	67	2.677	0.1018
	**Tumor**	105	110			102	113			115	100		
		**B7-H7**											
	**Normal**	58	62	0.2466	0.6195								
	**Tumor**	110	105										
**Grade**		**B7-1**				**B7-2**				**B7-DC**			
	**G1**	4	6	5.088	0.0786	5	5	24.25	<0.0001	4	6	23.34	<0.0001
	**G2**	58	77			45	90			46	89		
	**G3**	120	99			132	87			132	87		
		**B7-H1**				**B7-H2**				**B7-H3**			
	**G1**	4	6	6.082	0.0478	6	4	0.6263	0.7311	5	5	0.9699	0.6157
	**G2**	57	78			65	70			63	72		
	**G3**	121	98			111	108			114	105		
		**B7-H4**				**B7-H5**				**B7-H6**			
	**G1**	6	4	11.02	0.0041	4	6	4.189	0.1231	7	3	11.39	0.0034
	**G2**	82	53			59	76			81	54		
	**G3**	94	125			119	100			94	125		
		**B7-H7**											
	**G1**	7	3	7.721	0.0211								
	**G2**	78	57										
	**G3**	97	122										
**Race**		**B7-1**				**B7-2**				**B7-DC**			
	**Asian**	34	40	3.366	0.1858	36	38	4.929	0.0850	33	41	9.598	0.0082
	**White**	124	112			123	113			127	109		
	**Others**	3	8			2	9			1	10		
		**B7-H1**				**B7-H2**				**B7-H3**			
	**Asian**	31	43	3.846	0.1462	53	21	17.74	0.0001	29	45	5.105	0.0779
	**White**	126	110			103	133			125	111		
	**Others**	4	7			5	6			7	4		
		**B7-H4**				**B7-H5**				**B7-H6**			
	**Asian**	36	38	0.8691	0.6475	40	34	4.955	0.0840	35	39	2.486	0.2885
	**White**	118	118			119	117			118	118		
	**Others**	7	4			2	9			8	.3		
		**B7-H7**											
	**Asian**	34	40	2.773	0.2499								
	**White**	119	117										
	**Others**	8	3										
**Pathologic stage**		**B7-1**				**B7-2**				**B7-DC**			
	**Stage I**	21	32	3.886	0.2741	18	35	6.466	0.0910	18	35	7.912	0.0479
	**Stage II**	53	58			59	52			55	56		
	**Stage III**	81	68			78	71			84	65		
	**Stage IV**	20	17			20	17			18	19		
		**B7-H1**				**B7-H2**				**B7-H3**			
	**Stage I**	27	26	1.104	0.7760	17	36	8.69	0.0337	26	27	2.38	0.4973
	**Stage II**	51	60			62	49			58	53		
	**Stage III**	78	71			78	71			69	80		
	**Stage IV**	19	18			18	19			22	15		
		**B7-H4**				**B7-H5**				**B7-H6**			
	**Stage I**	32	21	6.208	0.1019	23	30	1.237	0.7441	26	27	7.085	0.0692
	**Stage II**	61	50			58	53			46	65		
	**Stage III**	67	82			76	73			79	70		
	**Stage IV**	15	22			18	19			24	13		
		**B7-H7**											
	**Stage I**	27	26	0.1337	0.9875								
	**Stage II**	54	57										
	**Stage III**	75	74										
	**Stage IV**	19	18										
**Tumor topography**		**B7-1**				**B7-2**				**B7-DC**			
	**T1**	5	14	7.035	0.0708	2	17	14.69	0.0021	4	15	12.11	0.0070
	**T2**	40	40			42	38			41	39		
	**T3**	81	87			82	86			78	90		
	**T4**	58	42			58	42			61	39		
		**B7-H1**				**B7-H2**				**B7-H3**			
	**T1**	5	14	8.817	0.0310	10	9	9.414	0.0243	5	14	4.774	0.1891
	**T2**	47	33			28	52			43	37		
	**T3**	77	91			92	76			85	83		
	**T4**	55	45			54	46			51	49		
		**B7-H4**				**B7-H5**				**B7-H6**			
	**T1**	14	5	10.4	0.0154	10	9	0.3051	0.9591	11	8	9.005	0.0292
	**T2**	48	32			40	40			35	45		
	**T3**	80	88			86	82			76	92		
	**T4**	42	58			48	52			62	38		
		**B7-H7**											
	**T1**	11	8	1.092	0.7790								
	**T2**	42	38										
	**T3**	80	88										
	**T4**	51	49										
**Lymph Node**		**B7-1**				**B7-2**				**B7-DC**			
	**N1**	51	62	3	0.3916	53	60	2.471	0.4805	49	64	3.196	0.3624
	**N2**	54	42			54	42			52	44		
	**N3**	35	39			34	40			38	36		
	**N4**	39	35			38	36			40	34		
		**B7-H1**				**B7-H2**				**B7-H3**			
	**N1**	55	58	1.47	0.6892	56	57	2.264	0.5195	62	51	1.984	0.5758
	**N2**	49	47			47	49			45	51		
	**N3**	41	33			42	32			38	36		
	**N4**	34	40			33	41			34	40		
		**B7-H4**				**B7-H5**				**B7-H6**			
	**N1**	57	56	5.578	0.1340	49	64	3.087	0.3783	55	58	1.825	0.6096
	**N2**	46	50			52	44			44	52		
	**N3**	45	29			39	35			39	35		
	**N4**	31	43			39	35			41	33		
		**B7-H7**											
	**N1**	57	56	1.213	0.7498								
	**N2**	44	52										
	**N3**	40	34										
	**N4**	38	36										
