# Supplementary material for: Comprehensive understanding of B7 family in gastric cancer: expression profile, association with clinicopathological parameters and downstream targets
Source: Int J Biol Sci. 2020 Jan 1;16(4):568–82. doi: 10.7150/ijbs.39769 (PMC6990920; doi:10.7150/ijbs.39769)
Supplement: Supplementary file 1 — Supplementary figures. [file ijbsv16p0568s1.pdf]

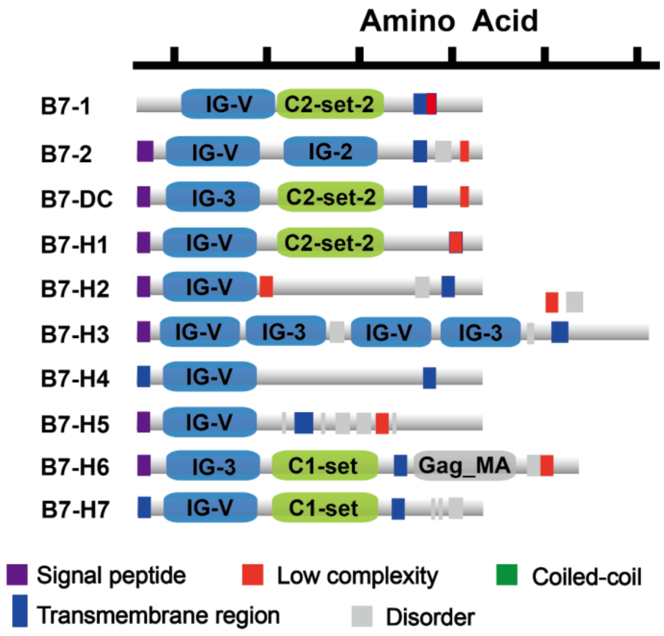

Amino Acid
B7-1
IG-V
C2-set-2
B7-2
IG-V
IG-2
B7-DC
IG-3
C2-set-2
B7-H1
IG-V
C2-set-2
B7-H2
IG-V
B7-H3
IG-V
IG-3
IG-V
IG-3
B7-H4
IG-V
B7-H5
IG-V
B7-H6
IG-3
C1-set
Gag_MA
B7-H7
IG-V
C1-set
Signal peptide
Low complexity
Coiled-coil
Transmembrane region
Disorder

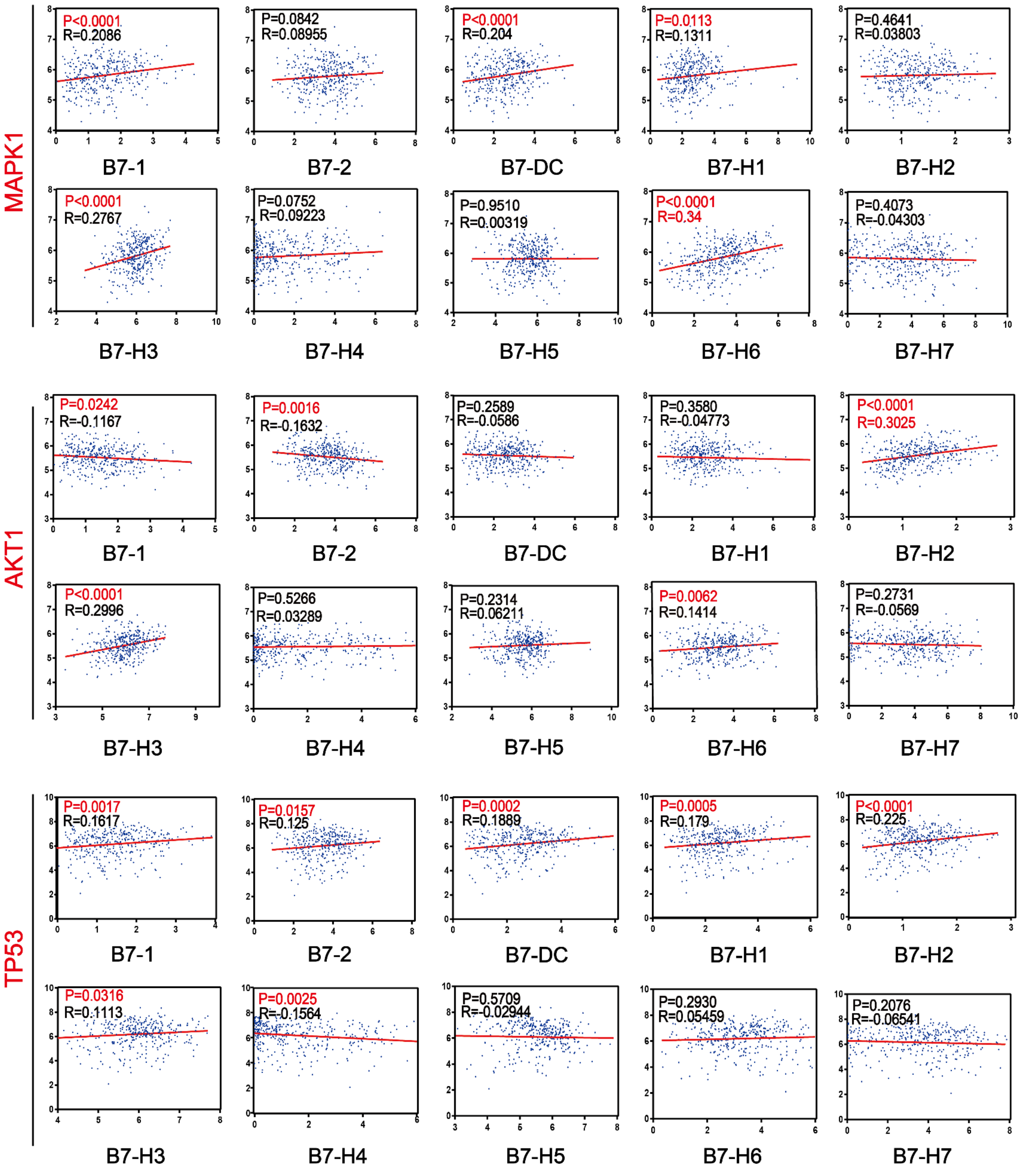
MAPK1
P<0.0001
R=0.2086
B7-1
P=0.0842
R=0.08955
B7-2
P<0.0001
R=0.204
B7-DC
P=0.0113
R=0.1311
B7-H1
P=0.4641
R=0.03803
B7-H2
P<0.0001
R=0.2767
B7-H3
P=0.0752
R=0.09223
B7-H4
P=0.9510
R=0.00319
B7-H5
P<0.0001
R=0.34
B7-H6
P=0.4073
R=-0.04303
B7-H7
AKT1
P=0.0242
R=-0.1167
B7-1
P=0.0016
R=-0.1632
B7-2
P=0.2589
R=-0.0586
B7-DC
P=0.3580
R=-0.04773
B7-H1
P<0.0001
R=0.3025
B7-H2
P<0.0001
R=0.2996
B7-H3
P=0.5266
R=0.03289
B7-H4
P=0.2314
R=0.06211
B7-H5
P=0.0062
R=0.1414
B7-H6
P=0.2731
R=-0.0569
B7-H7
TP53
P=0.0017
R=0.1617
B7-1
P=0.0157
R=0.125
B7-2
P=0.0002
R=0.1889
B7-DC
P=0.0005
R=0.179
B7-H1
P<0.0001
R=0.225
B7-H2
P=0.0316
R=0.1113
B7-H3
P=0.0025
R=-0.1564
B7-H4
P=0.5709
R=-0.02944
B7-H5
P=0.2930
R=0.05459
B7-H6
P=0.2076
R=-0.06541
B7-H7
